# Notes and Comments

**Published:** 1923-09

**Authors:** 


### NOTES AND COMMENTS.

### PAYMENT OF HOSPITAL STAFFS.

THE doctors and the hospitals have another year in which to make up their minds whether it is right or wrong that a percentage of payments made for hospital benefits should be passed into a fund at the disposal of the honorary medical staff, the Conference of the Representative Body of the British Medical Association having decided by a small majority to defer the further consideration of the subject until its next meeting. Standing by itself such a decision would have been rather timorous, but it is coupled with an instruction to the Council to ascertain from the medical staffs of all hospitals of not less than thirty beds "which of the various sources of income, if any, of a hospital should be required to make a contribution towards a staff fund." The division of opinion among doctors is probably greater than is suggested by the voting, but we find it difficult to believe that many of them regard the voluntary system as "played out," as is sometimes suggested. That great system, badly shaken as it was by the war and by the economic difficulties which followed, has lately shown signs of recovery too remarkable to be mistaken. Moreover, the voluntary system does not rest upon the non-payment of the doctors—the notion that it does springs from an entire misconception of the phrase. A change is taking place in the outlook of the hospitals, and if middle class people in considerable number are in future to use the hospitals it would be entirely reasonable that the medical staffs should be compensated for the new demands upon them. The subject needs to be dealt with cautiously, and there is no need to regret its postponement. When the reports from the hospitals are tabulated next March we shall be in a much better position to deal with it. Meanwhile let us not be unduly anxious about the voluntary system.

### ICE AS A LUXURY.

THE recent spells of torrid weather have once more produced complaints of the cost and scarcity of ice. It is not surprising that London icemaking machinery should for a week or two have been inadequate to the unexampled demand, but ice in London is always dear, partly, no doubt, because it is never very plentiful. In the country, if we except the greater towns, it is almost unknown at any time, and one of the outstanding trials of the American visitor is that, as often as not, he cannot obtain iced water. However sincerely we may sympathise with people whose accustomed comforts are suddenly cut off, it is likely enough that his health is improved by his enforced partial, or complete, abstinence. The constant absorption of iced drinks is not good for the human machinery, but there are uses for ice in hot weather which remove it far from the category of a luxury and make it a necessity. The man who drinks a liquid which has been "on the ice" is in happier case than he who swallows one in which large lumps of ice are floating. Tepid drinks on a grilling day are an abomination; so, in another sense, are drinks in which ice has been dissolved. It is never easy to be sure of the purity of the ice, and if that argument fails to appeal to the thirsty man, he may not be insensible to the fact that this excessive dilution is likely to spoil the flavour of what he is drinking, while the frequent freezing of the digestive apparatus is pretty sure to cause trouble some day. One of the most important, and legitimate, uses of ice is to keep food cool and fresh. Save in the country, England is not a land of cellars, but nothing is more "grateful and comforting" in the midst of heat than a liquid which has the natural freshness imparted by a good cellar. But, in any case, chilled drinks require to be used with caution.

### FOOD POISONING AND FOOD INFECTION.

As we have recently been through two spells of those alternating damp and hot, dusty days which are exactly suited to thorough distribution of germs among our daily foodstuffs, it may be well to call attention to a note of the result as supplied to us by one of London's large central laboratories. No sooner did the first hot spell begin than "food poisonings" cropped up all over the town. It has become customary popularly to consider food poisoning as implying only the "ptomaine" poisoning associated with putrefaction of food. Actually this habit tends to obscure the real point of one of our great public dangers—the germs carried by dust and flies to our food. There has of late been a positive outbreak of dysenteric troubles, fortunately mild and not associated, of course, with the true dysentery of the tropics but highly unpleasant and, in weaker people, dangerous nevertheless. It is, from the circumstantial as well as laboratory evidence, not a case of canned foods, botulism or "bad" foods, but just dust and dirt of the streets and countryside conveyed to our food. As usual at this time of the year, and largely from the same causes, the germs of typhoid and paratyphoid fevers, particularly the latter, have also shown renewed activity. It is true that we are daily making progress in discovering sources of food infection, and to realise this we have only to read the various publications of the Health Ministry; but as long as our food is so ruthlessly exposed to the dust and dirt of the streets and to the direct contact of so many but half-washed hands, so minor food poisonings, "abdominal influenzas," enteritis cases, or whatever we choose professionally to call them, will flourish and will take their toll of health and even of life itself among our less robust citizens.

### THE GLOUCESTER OUTBREAK.

THANKS to prompt and energetic measures of isolation, disinfection and vaccination the epidemic of smallpox at Gloucester, which at one time was so threatening, is over. How threatening it was we may judge from the fact that on a given date there were 254 cases actually in hospital. Happily, the character of the visitation was mild, but a mild epidemic of this terrible disease may at any moment become virulent, and it cannot be said that any epidemic of smallpox is mild until it is over. Before the outbreak only some 30 per cent. of the population of Gloucester was vaccinated; the proportion has now been nearly doubled. There have been roughly 2,000 cases of smallpox in the city since last December and the population being only 52,000 the percentage of seizures is very serious. Gloucester's nerves have been badly "rattled," and we trust that vaccinations will continue on a large scale until the number of the unprotected is negligible. There are many towns no better vaccinated than Gloucester was two or three months ago, and in such communities there is always danger—danger which may readily be extended to other places. Medical men would do well to be a little less shy in pressing upon their patients and others whom they can influence not merely the importance but the imperative duty of being vaccinated.

### HAND-IN-HAND.

AT the Annual Conference of the British Medical Association, held at Portsmouth, there was discussed, and agreed upon, a scheme of co-operation between that body and the Society of Medical Officers of Health. This scheme provides for direct representation of the Public Health Service in the governing bodies of the Association and direct representation of the Association in the Branches and on the Central Council of the Society of Medical Officers of Health. It also involves the relinquishment by the Society of Medical Officers of Health of independent medico-political action; the taking of such action will remain in the hands of the Association which represents all interests in the profession. It would be unwise to assume that the public interest took precedence over that of the profession in the proposals underlying this scheme of co-operation. But on broad public grounds this linking-up of the great forces of preventive and curative medicine, of members of the medical profession whose work is so intimately related, is pre-eminently the right step, and we congratulate the two bodies on arriving at so satisfactory an agreement.

### OCCUPATIONAL THERAPY IN HOSPITAL PRACTICE.

SEEING that most of our metropolitan hospitals are confronted with the perpetual difficulty of accommodating their convalescents sufficiently long and yet not unjustifiably reducing the space available for new-comers, the following American experiment is interesting. Dr. C. L. Vaux, in the *Long Island Medical Journal*, describes how, in a State Hospital, the average period of convalescence has been remarkably shortened by systematic occupational therapy. It is not that the occupation makes the fractured bone unite any quicker, but rather that the occupied patient is less irritable or complaining. Essentially he rests better, and a contented mind inevitably augments his physical recovery. Apathy and morbid introspection are reduced to a minimum. From his idle phantasies to surrounding realities is a step towards health, both in thought and action. Now, in a general way, all our hospitals attempt to give some kind of occupation to their convalescents. But the matter as often as not rests largely with the personal disposition of the sister and her staff. There is little or no official and organised scheme whereby the convalescent patients can be more rapidly "unhospitalised." Yet in America there is now probably no activity of the hospital which does not in some way or other enlist the help of "patient labour." Cleaning activities, sewing, working various machines, cooking, ironing and the rest all form obvious possible instances. But the American scheme goes farther. Excellent classes are organised so that really useful, skilled help may be given and be of practical value in the hospitals' economy. Interspersed with the work are all manner of well organised amusements, and, however long his stay, practically every patient rapidly becomes and remains a useful member of the institution. The matter is receiving so much attention on the other side of the Atlantic that those interested in English institutional management would do well to follow these developments closely.

### FEWER FLIES.

DESPITE the excessive heat of parts of July and August there has, happily, been a comparative scarcity of flies. Some districts have been freer than others, but the plague has, generally speaking, been less severe than in ordinary summers. The fact has little, if any, permanent significance. In the early days of summer there was a sufficiency of heavy rain to destroy the embryo fly in great numbers. Then there have been periods of "grateful coolness after heat" before the next hot spell which cannot have conduced to the longevity of flies, however favourable they may have been to the health of human beings. Numbers have thus been kept down, but there are still flies enough to carry heavy loads of infection wherever their wings may take them, and we may be sure that the plague is likely to be with us for many a day to come. So long as we store up heaps of garbage for a week or two at a time, instead of burning it daily, the fly will flourish. If only the fly were as abominable to look upon as the cockroach everybody would kill it at sight. Yet the cockroach, hateful as it is, does a certain amount of scavenging work in the intervals of its favourite occupation of gnawing through plaster, whereas the fly, whatever its species, is one of the most active disseminators of disease. Some day it will be exterminated, but the process of extermination will be slow.

### CONSOLIDATION OF HEALTH AND HOUSING LAWS.

WE referred last month to the introduction by the Earl of Onslow of a Bill consolidating the National Health Insurance Statutes. We may expect to see this useful and valuable measure passed into law in the Autumn Session. It is worthy of note that Mr. Neville Chamberlain announced in the course of the debates on the new Housing Act that a measure for the consolidation of all the Housing Acts was being prepared and would be introduced, he hoped, early next year. The new Housing Act contains various provisions simplifying and making uniform the existing law "for the purpose of facilitating the consolidation and of simplifying the machinery of the Housing Acts," and it is of course desirable that these simplifications should become law before any consolidating measure is introduced. There are, however, many other Statutes which urgently require consolidation. Among them it is sufficient to mention the vast number of Poor Law Statutes, the Public Health Acts, and Local Government Acts. The Rating Reform Bill, which it has been announced will be introduced by the Government next year, will doubtless lay the foundation for an Act consolidating the numerous and complicated Statutes which regulate the law of rating generally. The Burial Acts, the Acts relating to water, and many others, also require to be consolidated when opportunity offers. We trust that the Minister of Health, whose practical experience in administration everyone recognises, will map out a comprehensive programme of consolidating measures. A programme of this kind is not the sort of thing on which speeches are made at General Elections, but those who are interested in local government, and especially in matters relating to public health, know well its importance; and we hope to see within the next few years a great advance made in clarifying and simplyfying the hideous confusion of our present Statute Book.

### FAILURE TO NOTIFY TUBERCULOSIS.

In certain districts it has been found that more than 40 per cent. of the persons dying from tuberculosis had not been previously notified as suffering from that disease. This failure to perform a duty which has been incumbent upon all medical practitioners since 1913, has become so marked that the Minister has taken the unusual step of communicating direct with every practitioner in England and Wales on the subject. As the circular letter so issued points out, the prompt notification of all cases of tuberculosis is of importance in the interests of the community as well as of the patient. In this way only can full and early co-operation be secured between the medical practitioner, the Medical Officer of Health of the district and the Tuberculosis Officer, in order that all possible steps may be taken to prevent the spread of infection, and to insure that the patient receives the treatment best suited to his condition. We share the hope expressed by the Minister that local authorities will have no need to resort to legal proceedings to secure compliance with the Regulations. The machinery of notification is so essential a part of the public provision for attacking tuberculosis that no general practitioner can lightly afford to disregard his obligations in the matter.

### BLACK SMOKE AND GREY.

"The dirtiest country in the whole of Europe" is the cheerful way in which Lord Newton describes his native land. Happily he was referring not to the personal habits of his fellow-countrymen, but to the easy nonchalance with which they allow the pollution of the air they breathe. On the whole he is satisfied with the Government's Smoke Abatement Bill, which has been read a second time in the House of Lords, though he does not understand why the Government's own establishments should not come in. But Governments never like to run the risk of having to pay fines into their own exchequer. The measure certainly does not go as far as it might, but if, when it is passed, it really is enforced, it ought to do much towards clearing the atmosphere. Henceforward it will be the volume, rather than the colour, of the smoke emitted from factories which will come to judgment. Thus grey, or even white, smoke may be as culpable as black, but local authorities are left to prescribe the degree of density which constitutes a nuisance, which will probably mean one density at Bournemouth and a very different one at Birmingham. The penalties are greatly increased, and we hope that they will often be inflicted to the full on the great principle of encouraging the others. Perhaps, too, it would encourage the other Parliamentary draftsmen if they were paraded to witness the hanging of the author of this Bill, who, in four pages, seeks to legislate by nine-and-twenty references to existing Acts. We make bold to say that most, if not all, of these references were entirely unnecessary.

### RE-ASSURING THE ASSURED.

It is so plainly to the advantage of insurance companies that their policy-holders should live as long as possible that the motive underlying the proposal of two or three of them that they should give their assured the benefit of an annual free medical examination has much more in it than mere advertisement. The policy-holder would benefit likewise. He would be warned of the threatened onset of disease or would have it discovered in its early stages, while the value of his policy would be improved by the addition of the larger profits which would accrue from the lengthened life of a large number of assured persons. This, however, could only happen if these annual examinations became general, and the contingency may be put aside as a minor consideration. What really matters is the nipping of disease in the bud. In the affairs of health the world has been slow to learn that prevention is better than cure, and we are only now beginning to realise how vast and splendid is the prospect that lies before preventive medicine. There is everything to be said for a periodical overhaul. Many people visit the dentist regularly as a precaution; but how often do we hear of a doctor's patients doing likewise? The robust may shrug their shoulders and talk scornfully about "valetudinarians," but precautions of this kind may be as valuable at forty as at sixty. The outstanding characteristic of most of us is lack of wisdom, and in no direction do we show that lack more abundantly than in the calm *insouciance* with which we wait for the illness which, in the circumstances, is sure to come. Yet how vast is the mass of ill-health and actual disease which might be averted by a little enlightened selfishness!

### THE HUMAN CARRIAGE.

So considerable a time has elapsed since the human animal adopted the upright position that he might reasonably have been expected to perform rather well on two legs. But, as the recent discussion at the meeting of the British Medical Association reminds us, he is much less upright than he ought to be. The slouching gait and the pushed forward head are still exceedingly common. which is the more remarkable since most young men are inordinately addicted to games. Yet it may be doubted whether games are really the best training for an upright carriage. Military drill alone appears to be successful in that direction, as we may see in any street any day in the carriage of the young men who not so long ago passed through the army. On the whole, perhaps, women hold themselves better than men—their particular failure is about the feet, which, generally speaking, are too much turned out. Many centuries of trying to overcome the attrition of heavy clinging skirts may have something to do with this. That disadvantage is at an end now that skirts have become elementary in form and often singular in number. Detachments of girl guides march admirably, with grace and rhythm. Good health and a good carriage often go together, and it is certain that a slovenly gait deprives the vital organs of some of the advantages which nature intended them to enjoy.

### HELIOTHERAPY.

SO much has been heard lately of sunlight treatment such as is practised at Leysin or on our own South Coast that we read with interest in the *South African Medical Record* an account by Dr. Daneel of the application of these methods in South Africa. It is accepted now that heliotherapy is more effective at high than at low altitudes; and this is not due simply to the clearer air and stronger light and active rays. At high altitudes the circulating blood contains an increased number of red cells and more hæmoglobin, and has therefore a greater power to absorb oxygen from the air. Also the action of the lungs and hence the circulation of the blood there is more vigorous when in the higher lands. Apparently, however, it is difficult to find the exactly ideal conditions in South Africa in spite of her size and varied surface, for in places otherwise suitable the actual heat power of the sun is so great that the patients cannot be satisfactorily exposed for any length of time. Nevertheless the Free State, Basutoland, and many of the eastern coast areas provide most of the conditions, and the author has had undoubted success with his methods. These, we note, are commendably thorough, as the sunlight is not asked to work cures unaided. Whenever there is possibility that the patient's resistance and recovery can be promoted by use of curative sera, vaccines or drug treatment, these are carefully utilised at the same time and every case undergoes clinical and laboratory examination before the treatment phase commences. In short, every usual means is employed, and added to all is heliotherapy —an eminently sound proceeding.

### THE FUTURE OF BETHLEM HOSPITAL.

THE report of Mr. Lionel Faudel-Phillips, the treasurer of Bethlem Royal Hospitals, is a document of annual interest, not merely of annual custom. In the latest he announces changes of importance both to the history of the foundation and in themselves. The present building, sounder in structure than convenient in design, is now 108 years old. Moreover, the grounds of five acres, generous of space though they once were, are now too small for their varied purposes. Inside the building the nurses on the female side are still housed in wards, a hospital practice once the rule but now clearly out of date. These admissions pre face a hint of "far-reaching" schemes, the explanation of which might be premature though a shrewd idea of them may readily be formed. As Mr. E. W. Morris found in connection with patients' payments at the London Hospital, the relatives of patients in Bethlem are found often to strain unduly their own resources to contribute to the cost of maintaining their dependants in the hospital. Bethlem, too, feels the lack of suitable candidates for the nursing staff, despite the advantages of salary, leave, annual and weekly, and pensions for those past work, offered by the hospital. Being well endowed, Bethlem does not complain unduly of debt, or even want of capital. There is no more remarkable tribute to the voluntary system than the prosperity of its most ancient institutions. Bethlem has existed for almost 700 hundred years, which encourages us to believe that the hospitals which now groan beneath their overdrafts have only to wait, in loyalty to their best traditions, to be equally independent and strong. They are all, however old, by comparison the younger institutions, and history proves that time is on their side and cuts out all excuse for faint-heartedness.

### DENTAL CARIES.

IT is a curious fact that things most constantly under our notice are often those that are least well understood. The simple decay of teeth is a typical example. Dental caries is a universally prevalent disease; its cause is quite unknown. Also, in spite of the claims of tooth paste manufacturers, we do not in the least know how to prevent it, except in the most general way. A subject in this state is inevitably surrounded by theories, and with caries these fall into two groups. The first seeks to explain the damage to the teeth as occurring secondarily to constitutional troubles such as malnutrition, rickets, deficiency of vitamines, etc. The second gives as a cause the locally destructive action of bacteria or acids in the mouth. Out of this mass of indeterminate evidence it appears likely that the truth will emerge as a combination of both explanations, for there seems little doubt that some people's teeth are much more liable to decay than others, in spite of the fact that they all may be living under exactly the same conditions, swallowing the same dust, eating the same food and subjecting their dental equipment generally to identical wear and tear. This would suggest that something in the structure of the tooth may be deficient; its development, microscopically considered, may be imperfect, and for this reason the local destructive agencies within the mouth are allowed more readily to act. It is probably not a case of seeking for this or that bacterium or eschewing some particular food or dentifrice, but rather of amending the details of a growing child's upbringing. A great deal has been done in this respect, but research into the dental development of the growing child and the means to improve it are among our greatest national needs.

